# A Rare Case of Early Left Ventricular Thrombus Development After Off-Pump Coronary Artery Bypass Grafting for Unstable Angina Pectoris With Reduced Left Ventricular Ejection Fraction

**DOI:** 10.7759/cureus.74938

**Published:** 2024-12-01

**Authors:** Yojiro Machii, Yuki Hayashi, Atsushi Harada, Yuzo Ezawa, Masashi Tanaka

**Affiliations:** 1 Department of Cardiovascular Surgery, Nihon University School of Medicine, Tokyo, JPN

**Keywords:** anticoagulant therapy, heart failure with reduced ejection fraction, left ventricular thrombus, myocardial infarction, off-pump coronary artery bypass grafting

## Abstract

Left ventricular (LV) thrombus is a serious complication of myocardial infarction (MI) that can lead to a fetal systemic embolism. Although coronary artery bypass graft surgery (CABG) after MI is widely performed, to our knowledge, there are no reports of LV thrombus in the early postoperative period. Here, we report a rare case of a 70-year-old man who underwent off-pump coronary artery bypass grafting (OPCAB) for unstable angina pectoris with reduced left ventricular ejection fraction (LVEF). An LV thrombus (13 mm × 12 mm) was incidentally discovered on an echocardiography 7 days after OPCAB. We administered an oral anticoagulant (OAC) and observed resolution of the thrombus five months post-surgery. In the present case, factors such as low LVEF, infarcted lesion at the apex, and hypercoagulable state due to systemic inflammation after CABG likely contributed to LV thrombus development. This case highlights that LV thrombus is a potential complication early after CABG; thus, careful follow-up is required during the early postoperative period. Prophylactic anticoagulant administration in the early postoperative period should be considered for patients at particularly high risk for LV thrombus.

## Introduction

Left ventricular (LV) thrombus is a frequent complication of acute anterior wall myocardial infarction (MI) with reduced left ventricular ejection fraction (LVEF) [1,2]. LV thrombus is also a complication of primary percutaneous coronary intervention (PCI) [2,3]; however, to our knowledge, there are no reports of LV thrombus after coronary artery bypass grafting (CABG). Herein, we report a case of early LV thrombus development after off-pump coronary artery bypass grafting (OPCAB).

## Case presentation

A 70-year-old man undergoing hemodialysis presented to our emergency department with a sudden onset of severe dyspnea lasting for 3 hours. He had type 2 diabetes mellitus and chronic kidney disease (CKD) secondary to diabetic nephropathy. In addition, he was previously admitted to another hospital for the exacerbation of chronic heart failure (CHF). At that time, he had undergone percutaneous plain balloon angioplasty for a left main trunk (LMT) to the left anterior descending artery (LAD) lesion 14 months earlier, resulting in full perfusion (thrombolysis in myocardial infarction flow grade 3). However, chronic complete occlusion of the left circumflex artery (LCX) was not amenable to wire passage due to technical difficulty, and the patient was scheduled for watchful waiting.

On admission, he was in acute distress with a systolic blood pressure of 179 mmHg and diastolic blood pressure of 122 mmHg, pulse rate of 124 beats per minute, respiratory rate of 30 breaths per minute, temperature of 35.9 °C, and percutaneous oxygen saturation of 85% under oxygen administration (fraction of inspiratory oxygen: 0.8). During examination, significant wheezing and course crackle sounds were identified in both lungs and bilateral leg pitting edema was observed. Blood tests revealed negative results for creatinine kinase at 200 U/L (CK-MB, 8 U/L). N-terminal pro-brain natriuretic peptide was significantly high (29064 pg/ml). His hemoglobin A1c level was high (8.6%) (Table 1), and the patient was at high risk for perioperative infection.

**Table 1:** Summary of blood test results on admission CK: creatine kinase, CK-MB: creatine kinase MB isozyme, NT-proBNP: N-terminal pro-brain natriuretic peptide

Blood test	Values	Reference range
CK	200U/L	Men: 59-248U/L, Women: 41-153U/L
CK-MB	8U/L	0-12U/L
HbA1c	8.6%	4.6-6.2%
NT-proBNP	29064 pg/ml	0-125 pg/ml

**Figure 1:**
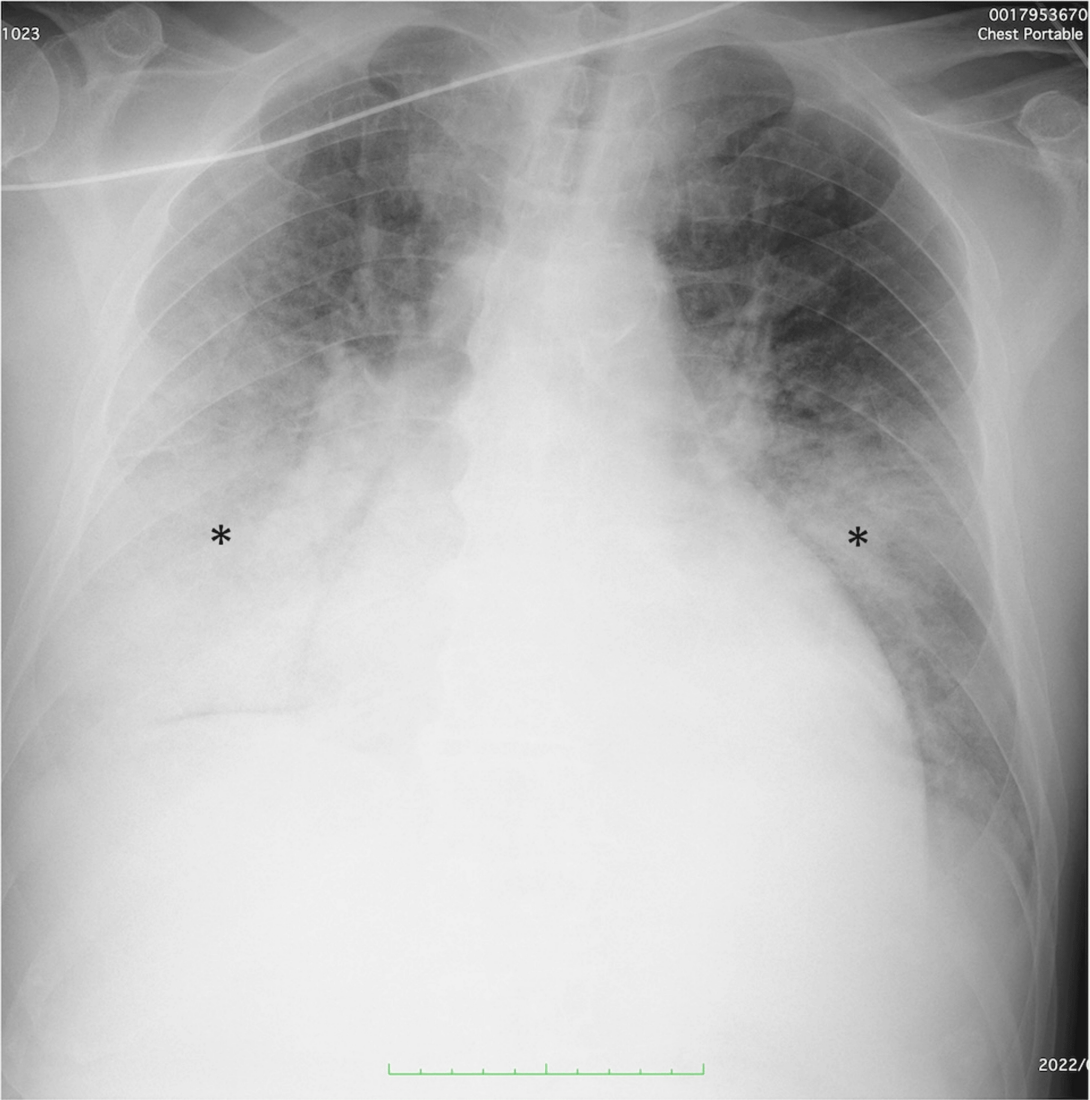
Chest X-ray on admission Chest X-ray on admission showing significant bilateral alveolar edema and pleural effusion (asterisk)

Chest radiography revealed significant bilateral alveolar edema and pleural effusion (Figure 1).

**Figure 2:**
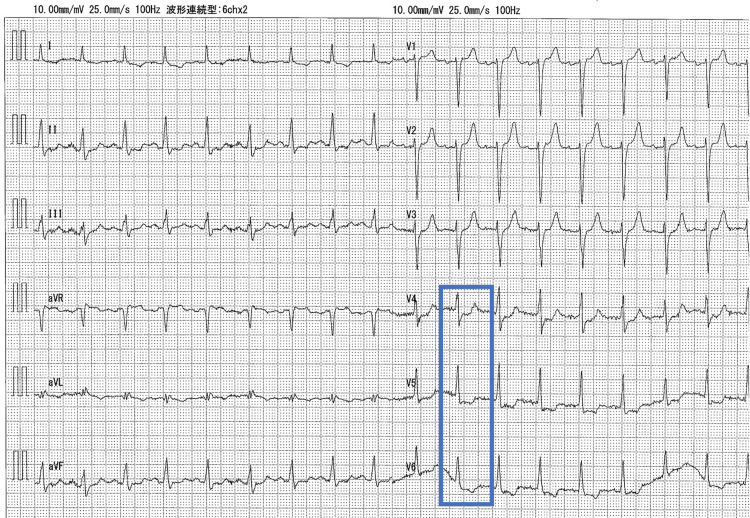
Electrocardiogram on admission Electrocardiogram showing sinus tachycardia and ST-segment depression in leads V4-V6 (blue). ST-segment elevation was not observed.

Electrocardiography revealed sinus rhythm and ST depression in the lateral leads (V4-6) (Figure 2).

Transthoracic echocardiography (TTE) revealed a reduced LVEF (28.7%), which was newly decreased compared to 3 months prior (LVEF: 45%) but no significant asynergy. LV wall motion was generally hypokinetic, with severe hypokinesis from the inferior to the posterior wall. There was no evidence of valvular disease, LV thrombus, or apical aneurysm that could have caused the LV thrombus (Video 1).

**Video 1:** Preoperative transthoracic echocardiogram (apical 2 chamber view) Left ventricular (LV) wall motion was generally hypokinetic, with no apical aneurysm or LV thrombus. Simpson's method for LV ejection fraction calculation was used.

**Figure 3:**
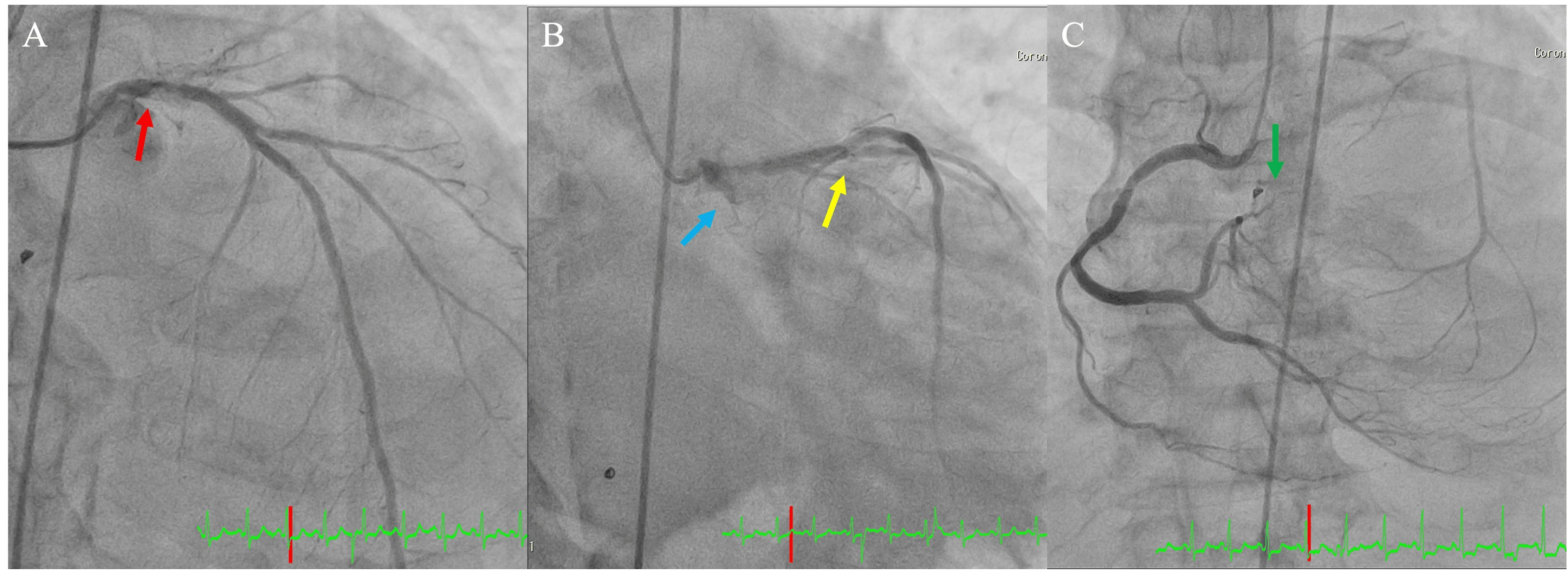
Coronary angiogram Coronary angiogram (A) (B)(C) showing three-vessels disease (left main trunk #5:75% (red arrow), left anterior descending #6-7:75% (yellow arrow), left circumflex #11:100% (blue arrow), right coronary artery #4 atrioventricular branch: 100% (green arrow))

Based on these findings, the patient was diagnosed with an acute exacerbation of CHF. Non-invasive positive pressure ventilation was immediately initiated, and diuretics were administered. On day 5 of admission, coronary angiography revealed three-vessel disease (LMT #5:75%, LAD #6:75%, LCX #11:100%, and right coronary artery #4 atrioventricular branch: 100%) (Figure 3).

**Figure 4:**
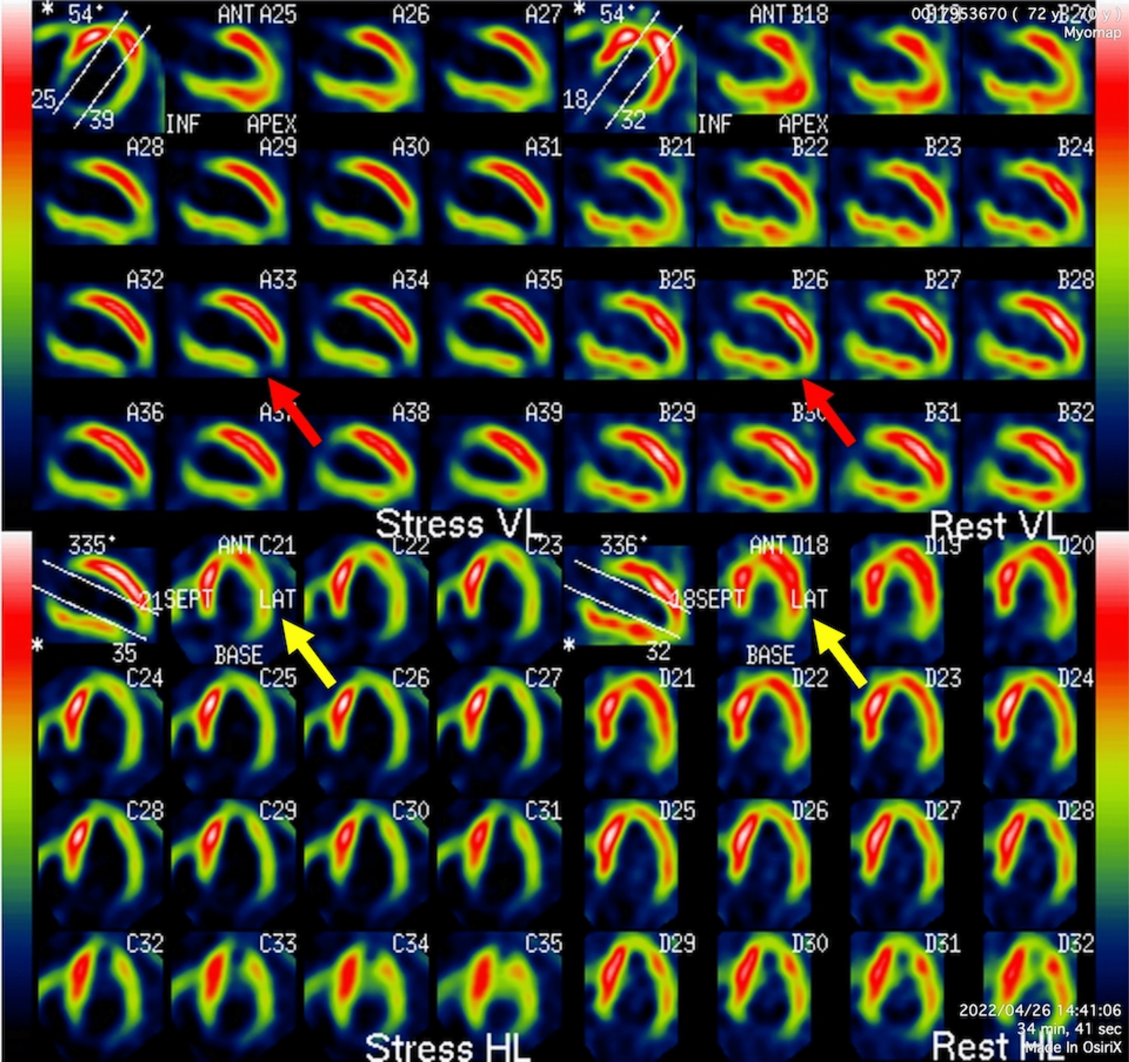
Myocardial single photon emission computed tomography Myocardial single photon emission computed tomography shows reduced blood perfusion at the apex both during exercise and at rest (red arrow). In addition, reduced blood perfusion is observed during exercise in the LAT (yellow arrow). Left ventricular myocardium other than the apex is shown to have viability because perfusion is maintained at rest (red and yellow arrows). The total perfusion deficit is 13%. VL: vertical long, HL: horizontal long, LAT: lateral wall

The patient responded well to treatment. To assess the severity and extent of myocardial damage and ischemia, myocardial single-photon emission computed tomography was performed, which showed a small prior MI in the apical wall and a large amount of ischemia in the entire lateral wall (Figure 4).

Since the LV myocardium remained viable except for the apex and the patient had three-vessel disease for which PCI was technically difficult, the decision was made to perform CABG. OPCAB was also selected because of the patient’s complex comorbidities, including hemodialysis. The Society of Thoracic Surgeons Risk and EuroSCORE II scores of operative mortality were 5.00% and 6.44%, respectively.

**Figure 5:**
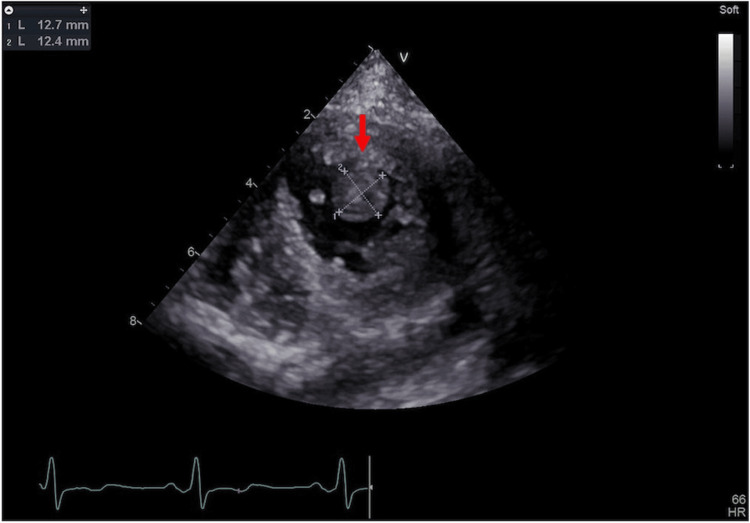
Left ventricular thrombus in the apex (parasternal short axis view apical level) Transthoracic echocardiography on postoperative day 9 showing a 13 mm × 12 mm poorly mobile isoechoic lesion in the apex of the left ventricle (red arrow)

Surgery was performed via a median sternotomy. Unfractionated heparin was administered intraoperatively to achieve an activated clotting time of 250-300 seconds. The bypass targets were the LAD and posterior lateral branch of the LCX, and the grafts were the left internal thoracic artery and saphenous vein. Intraoperative transesophageal echocardiography, routinely performed to observe mitral regurgitation during OPCAB, showed no thrombus in the left ventricle. The surgery was completed uneventfully, and the patient was extubated on the same day. On postoperative day (POD) 1, biaspirin at 100 mg/day was administered to prevent graft occlusion. Continuous renal replacement therapy for fluid management was initiated on POD 2 with nafamostat mesylate as an anticoagulant. On POD 9, TTE was performed to evaluate postoperative cardiac function; incidentally, a 13 mm×12 mm poorly mobile isoechoic lesion was found in the apex of the left ventricle (Figure 5, Video 2). The LVEF improved marginally to 32%. At this point, hypercoagulability after CABG and reduced LVEF were considered factors in LV thrombus.

**Video 2:** Echocardiogram on postoperative day 9 (parasternal short axis view apical level) Transthoracic echocardiography on postoperative day 9 showing a 13 mm × 12 mm poorly mobile isoechoic lesion in the apex of the left ventricle

**Figure 6:**
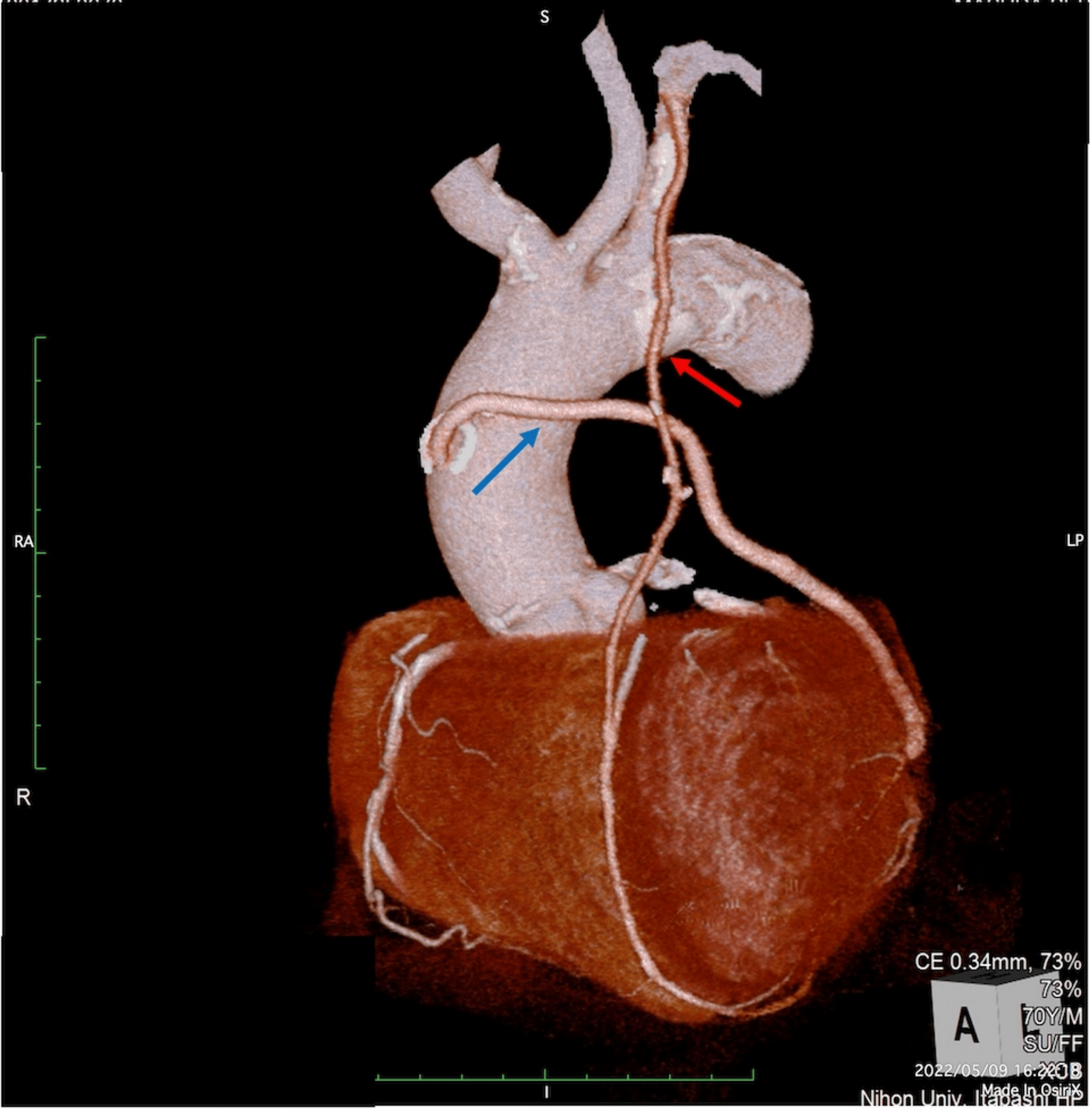
Coronary computed tomography angiography after coronary artery bypass grafting Coronary computed tomography angiography with contrast media showing patent grafts (left internal thoracic artery (red arrow) and saphenous vein (blue arrow))

After a full discussion with our Heart Team, a vitamin K antagonist (VKA) was administered to achieve a prothrombin time-international standard ratio of 2 to 3. Direct oral anticoagulants were not available due to CKD. On POD 11, computed tomography coronary angiography revealed that all grafts were patent (Figure 6).

**Figure 7:**
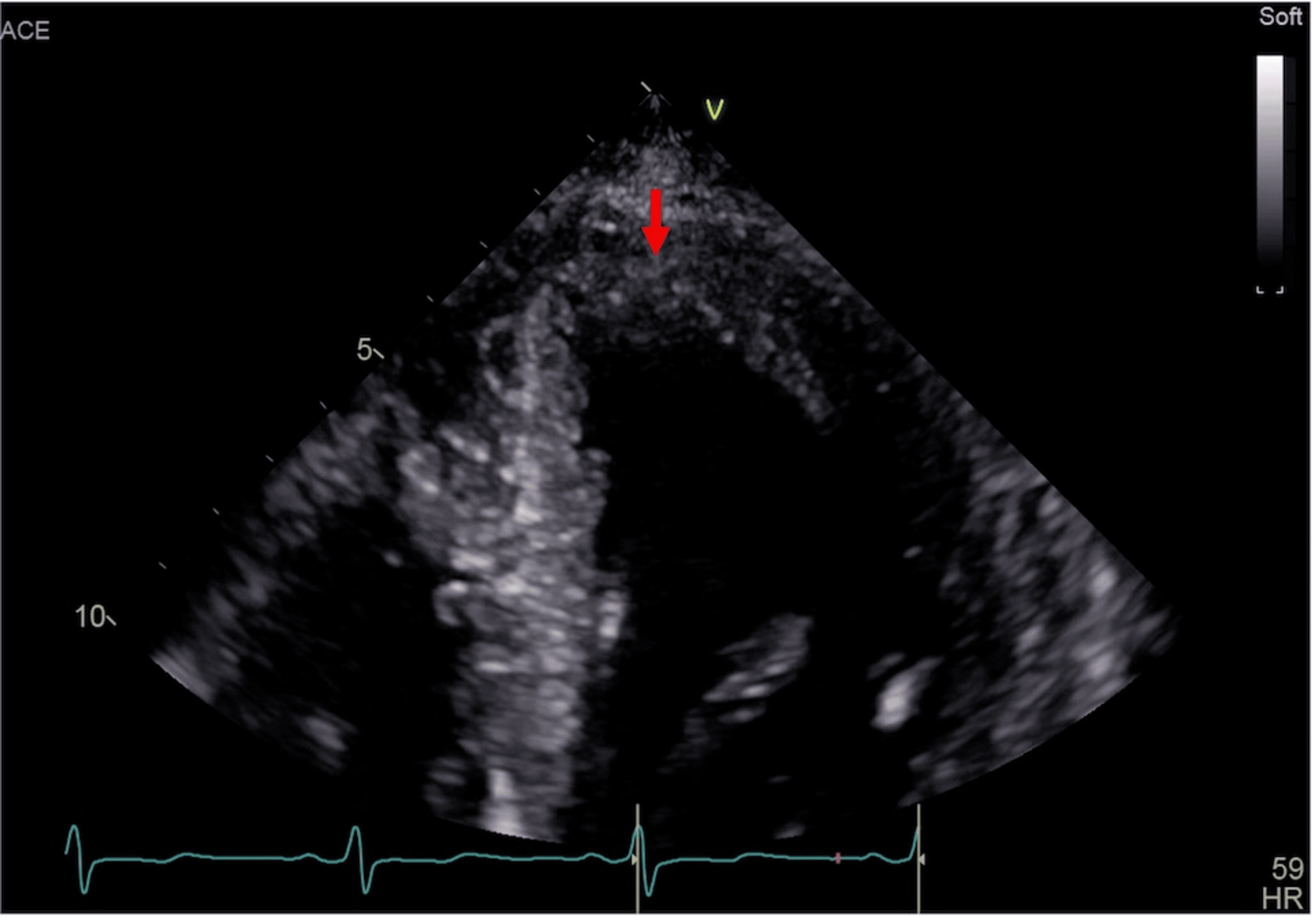
Disappearance of left ventricular thrombus (parasternal short axis view apical level) Transthoracic echocardiography performed five months after the surgery showing the disappearance of the thrombus (red arrow)

We performed TTE again during hospitalization, but the LV thrombus remained unchanged, and the patient was discharged on POD 26. The plan was to continue echocardiographic monitoring after discharge. VKA administration was maintained in an outpatient clinic, and TTE performed five months postoperatively confirmed that the thrombus disappeared (Figure 7, Video 3).

**Video 3:** Echocardiogram on five months postoperatively (parasternal short axis view apical level) Transthoracic echocardiography performed five months after the surgery showing the disappearance of the thrombus

No embolic events were observed. We plan to observe a recurrence of LV thrombus with TTE about once a year, but at this time there has been no recurrence.

## Discussion

LV thrombus is a serious complication of MI, and thromboembolic events can be devastating. Based on Virchow’s triad of thrombogenesis, the three major causes of LV thrombus are: blood stasis from LV dysfunction, endothelial injury from MI, and hypercoagulability triggered by inflammation [4,5]. LV thrombus is more prevalent after anterior wall ST-elevation MI, especially at the apex and in those with reduced LVEF [1]. It also occurs in non-recent MI, ischemic cardiomyopathy, and dilated cardiomyopathy [4]. In the present case, the patient had low preoperative LV function (LVEF, 28.7%), and the presence of an infarct lesion in the apex, as shown by single photon emission computed tomography (SPECT), which may have led to LV thrombus development. In addition, CABG, performed with and without cardiopulmonary bypass, causes a systemic inflammatory response and alterations in the normal mechanisms of hemostasis. Although OPCAB has been shown to protect against prothrombotic patterns during the intraoperative period [6], Edelman et al. reported that owing to both an increase in coagulation with elevation of fibrin generation and impairment of fibrinolytic potential (similar to on-pump CABG), until 10 days after OPCAB, patients remain in a prothrombotic state [7]. Based on these findings, we can assume that the patient was at an increased risk of developing an LV thrombus. The prophylactic administration of heparin and other anticoagulants for hypercoagulable states after CABG remains controversial. Edelman et al. reported that OPCAB and prophylactic anticoagulation should be considered in patients with preoperative MI [7]. We believe that anticoagulation should be used positively in the short postoperative period in patients at high risk of postoperative LV thrombus, as in this case, while considering the risk of postoperative bleeding.

Treatment of an LV thrombus immediately after cardiac surgery is very distressing considering the risk of bleeding due to anticoagulants and the invasiveness of surgical excision. Considering mobility and protrusion as the most important risk factors for LV thrombus-induced embolism [4], embolism was considered unlikely in this case, and oral anticoagulants (OAC) were the preferred treatment option over surgical excision. Although three months of OAC is recommended for the treatment of LV thrombus after acute MI, surgical excision is also tolerated to a limited extent in cases of embolism despite anticoagulation [4]. We believe that OAC was the appropriate choice, especially since this patient was at high risk for mediastinitis due to diabetes, and repeated median sternotomy was not advisable.

## Conclusions

This case demonstrates that anticoagulant administration to prevent LV thrombus should be considered in the early CABG postoperative period, especially for patients at high risk of LV thrombus. In addition, OAC administration should be considered for LV thrombus, which is thought to have a low embolism risk. Further studies comparing the timing and type of anticoagulant initiation after CABG are needed.
